# Improving Blood Pressure Control Through A Technology-Based Hypertension Self-Management Intervention

**DOI:** 10.1093/geroni/igaf122.3964

**Published:** 2025-12-31

**Authors:** Carolyn Still, Parishma Guttoo, Rania Aljohani, Siobhan Aaron, Carla Harwell

**Affiliations:** Case Western Reserve University, Cleveland, Ohio, United States; Case Western Reserve University, Cleveland, Ohio, United States; Case Western Reserve University, Cleveland, Ohio, United States; Case Western Reserve University, Cleveland, Ohio, United States; University Hospitals Cleveland Medical Center, University Hospitals Cleveland Medical Center, Ohio, United States

## Abstract

Hypertension affects 48% of U.S. adults and remains the leading cause of cardiovascular disease (CVD), with smaller demographic groups experiencing the highest rates and adverse health burden. Evidence suggests that the integration of digital health and technology-based interventions may offer strategies to self-management engagement, promote adherence to treatment regimens, and sustain lifestyle modifications, especially for populations experiencing despair associated with hypertension. However, evidence on the effectiveness of such technologies in African Americans (AA) remains sparse. This study investigates the effectiveness of a refined, multi-component technology-based intervention—OPTIMA-BP (OPtimizing Technology to Improve Medication Adherence and BP Control; n = 63) vs a Wait-list control group (n = 56) using a prospective, randomized controlled trial. The intervention integrates technology with tailored education and clinical support to enhance hypertension self-management. The sample comprised AA adults (mean age 63), diagnosed with hypertension, taking at least two antihypertensive medications, and owning a smartphone. Data was collected to assess demographic characteristics, health behaviors, medication adherence, technology adoption, and use. The primary outcome was systolic BP < 130 mmHg after 6 months. At baseline, the mean systolic BP for the intervention group was 137.89 mmHg (SD = 14.64), compared to 136.70 mmHg (SD = 13.73) for the Wait-list group. At 6 months, a greater reduction of systolic BP was noted in the intervention group, a decrease of 7.07 mmHg vs 1.93 mmHg for the Wait-list group. Preliminary findings suggest that technology-based interventions could be effective in improving hypertension self-management and BP control in the AA population.

